# Prevalence of diabetes mellitus and the performance of a risk score among Hindustani Surinamese, African Surinamese and ethnic Dutch: a cross-sectional population-based study

**DOI:** 10.1186/1471-2458-8-271

**Published:** 2008-08-01

**Authors:** Navin R Bindraban, Irene GM van Valkengoed, Gideon Mairuhu, Frits Holleman, Joost BL Hoekstra, Bob PJ Michels, Richard P Koopmans, Karien Stronks

**Affiliations:** 1 Department of Social Medicine, Academic Medical Centre of the University of Amsterdam, Amsterdam, The Netherlands; 2 Department of Internal Medicine, Academic Medical Centre of the University of Amsterdam, Amsterdam, The Netherlands

## Abstract

**Background:**

While the prevalence of type 2 diabetes mellitus (DM) is high, tailored risk scores for screening among South Asian and African origin populations are lacking. The aim of this study was, first, to compare the prevalence of (known and newly detected) DM among Hindustani Surinamese, African Surinamese and ethnic Dutch (Dutch). Second, to develop a new risk score for DM. Third, to evaluate the performance of the risk score and to compare it to criteria derived from current guidelines.

**Methods:**

We conducted a cross-sectional population based study among 336 Hindustani Surinamese, 593 African Surinamese and 486 Dutch, aged 35–60 years, in Amsterdam. Logistic regressing analyses were used to derive a risk score based on non-invasively determined characteristics. The diagnostic accuracy was assessed by the area under the Receiver-Operator Characteristic curve (AUC).

**Results:**

Hindustani Surinamese had the highest prevalence of DM, followed by African Surinamese and Dutch: 16.7, 8.1, 4.2% (age 35–44) and 35.0, 19.0, 8.2% (age 45–60), respectively. The risk score included ethnicity, body mass index, waist circumference, resting heart rate, first-degree relative with DM, hypertension and history of cardiovascular disease. Selection based on age alone showed the lowest AUC: between 0.57–0.62. The AUC of our score (0.74–0.80) was higher than that of criteria from guidelines based solely on age and BMI and as high as criteria that required invasive specimen collection.

**Conclusion:**

In Hindustani Surinamese and African Surinamese populations, screening for DM should not be limited to those over 45 years, as is advocated in several guidelines. If selective screening is indicated, our ethnicity based risk score performs well as a screening test for DM among these groups, particularly compared to the criteria based on age and/or body mass index derived from current guidelines.

## Background

Type 2 diabetes mellitus (DM) is one of the most common chronic diseases the world over, and the number of people with DM has risen sharply in recent years [1]. In the United Kingdom (UK), DM affects almost 1.8 million people, representing 3% of the population. There are also up to a million people with undiagnosed (asymptomatic) DM [2]. Other European countries report similarly high figures [3]. Individuals with DM are at high risk for cardiovascular disease (CVD). Adequate treatment of DM and associated risk factors such as hypertension and dyslipidemia greatly reduces the risk of complications.

While it is argued that there is no justification for universal screening for diabetes, there is strong support for screening and early treatment among population subgroups where DM is common and CVD risk is high [4]. Glucose levels are likely to be elevated for 10 years before DM is diagnosed [5]. This has led to recommendations for selective screening for DM by the American Diabetes Association (ADA) and Diabetes UK [6,7]. Current guidelines include questionnaires based on risk factors (e.g. age ≥ 45 years, BMI > 25) or the use of more complex risk scores which require invasive specimen collection [8-12].

These guidelines have been developed and tested particularly among populations of white European origin. This raises the question of whether they are valid for other ethnic groups as well, in particular populations of South Asian and African origin in Europe. As the prevalence of DM is higher in these groups, it may affect the efficiency of screening. For instance, while type 2 DM in white Europeans usually appears over the age of 40, it often appears before the age of 40 among South Asians and African origin populations [2,13-16]. In addition, the association between DM and its determinants might vary between ethnic groups.

The limited evidence indeed indicates that a risk score for DM developed for the white population is less efficient among South Asians and Africans [17]. Because of this, some guidelines recognize the necessity of adapting screening programs to ethnicity. The ADA questionnaire includes a question about ethnicity, and suggests screening before the age of 45 among specific groups [18]. However, to our knowledge, no tailored risk score for DM has been developed specifically for populations of South Asian or African origin. Moreover, it is not yet known at what age screening for DM should start, and which selection criteria are most efficient for these ethnic groups. Thus, the aim of our study was to provide information needed to optimize screening criteria for DM among different ethnic groups, e.g. Hindustani and African Surinamese migrants. Given the similarities in geographic and ethnic origin, it is expected that Hindustani Surinamese have much in common with South Asian migrants, and that African Surinamese are very similar to migrants of Afro-Caribbean ancestry in the United Kingdom (UK).

First, we determined the prevalence of known and newly detected DM in Hindustani Surinamese, African Surinamese, and ethnic Dutch (Dutch) in the Netherlands in two age groups: 35 to 44 and 45 to 60 years. Second, we developed a new risk score, based on ethnicity and biomedical risk factors that do not require invasive specimen collection in clinical practice. Finally, we evaluated the performance of that risk score and compared it with the current criteria derived from guidelines.

## Methods

**Figure 1:**
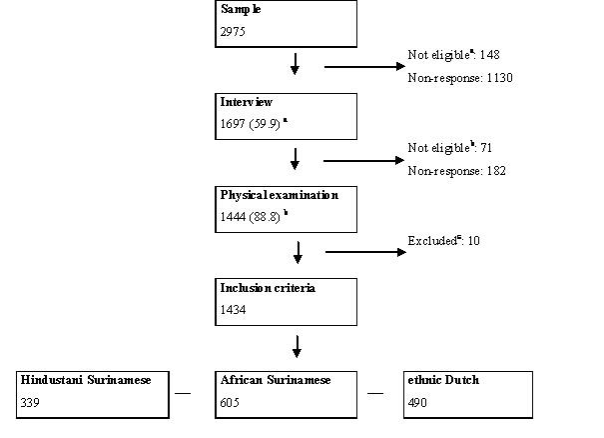
**Flow chart of the inclusion into the study**. ^a ^Persons who had moved, were deceased or could not be reached at the registered address were not considered as potential participants. ^b ^Only persons of Hindustani Surinamese, African Surinamese or ethnic Dutch origin were invited for the physical examination (n = 1626). Javanese or Chinese Surinamese persons and persons with missing ethnicity were excluded (n = 71). ^c ^Persons without a fasting plasma sample (n = 10) or were excluded.

The study population consisted of participants in the SUNSET study (Surinamese in the Netherlands: Study on health and Ethnicity) [19]. In 1975, almost half the population of the former Dutch colony Surinam migrated to the Netherlands. Approximately 80% of these Surinamese immigrants in the Netherlands are Hindustani ('South Asian', originally from the Indian sub-continent) or African (mixed African, Indian and European, but predominantly of African origin). The SUNSET study is based on a random sample of 2975 individuals, aged 35 to 60 years of age, drawn from the approximately 389000 ethnic Dutch (Dutch) and 72000 Surinamese listed in the Amsterdam population register (figure 1). For the sampling procedure, persons who were born in the Netherlands and whose parents were both born in the Netherlands were presumed to be Dutch. Persons of whom both parents were born in Surinam and persons who were born in Surinam and who had at least one parent who was born in Surinam were presumed to be Surinamese participants.

Between 2001 and 2003, all persons in the sample were approached for face-to-face, structured interviews by trained interviewers who had been matched by sex and presumed ethnicity. The interview included questions on self-identified ethnicity, migration history, demographic variables, lifestyle, and health status. If information on the self-identified ethnicity of the individual was lacking, the origin of the mother, the father and the mother's ancestors were used to classify participants.

The overall response to the interview was 60% (figure 1). Participation rates were higher among women than among men. In addition, participants in the interview were more likely to be married and living with a partner and/or children, and to live in a less urban area (address density of 1500–2500 addresses/km2 vs. ≥ 2500) as compared to non-participants (both non-response and not eligible). However, the absolute and relative differences between participants and non-participants for these characteristics were small and reported trends were similar across ethnic groups (data not shown).

Participants of Hindustani Surinamese, African Surinamese or Dutch origin were also invited for a physical examination at a local health care centre. During the examination, trained physicians recorded the following characteristics: weight in light clothing on a SECA mechanical scale to the nearest 200 grams and height to the nearest 0.01 meter by wall tape measure. Waist circumference midway between the lower rib margin and the iliac crest and hip circumference at the maximum point over the greater trochanters were determined to the nearest 0.01 meter by tape measure. After the subjects had emptied their bladder and had been seated for at least 5 minutes, blood pressure and resting heart rate measurements were obtained from each subject's arm at heart level using an OMRON-M4 semi-automatic sphygmomanometer with an appropriate-sized cuff. All anthropometric measurements were obtained twice and the means (rounded off to the nearest integer) were used for analysis. Fasting glucose (HK/Glucose-6-P dehydrogenase test; Roche Diagnostics, In), high density lipoprotein cholesterol (HDL, Homogenous enzymatic colorimetric test; Roche Diagnostics, In) and triglyceride (GPO-PAP Enzymatic test; Roche Diagnostics, In) levels were determined in serum samples obtained at the time of the physical examination. DM was defined as fasting glucose ≥ 7.0 mmol/l and/or self-reported DM, excluding the self-reported diagnoses of gestational diabetes.

The SUNSET-study was approved by the Institutional Review Board of the Academic Medical Centre of the University of Amsterdam, and carried out in compliance with the Helsinki Declaration. All participants provided a written informed consent.

In the present analysis, we included participants who had participated in both the interview and the physical examination. Of all participants in the interview, 71 were excluded due to missing information on self-identified ethnicity, 182 persons were excluded because they had not undergone the physical exam and 10 persons because fasting glucose measurements were not available (non-response for blood sample). As compared to those who were left in the study, those excluded were similar with regard to gender, self-reported DM and self-rated health (data not shown).

In total, 1434 participants remained in the study, divided into 339 Hindustani Surinamese, 605 African Surinamese, and 490 Dutch (figure 1). Of the Hindustani participants 98.8% were born in Surinam, 99.4 had two parents who were born in Surinam and 92.1% had two parents who were of Hindustani origin. Of the African Surinamese participants, 99.2% were born in Surinam and 99.5% had two parents who were born in Surinam. Moreover, 79.3% of the African Surinamese had two parents who were of African origin.

Characteristics of the ethnic groups were described using means or proportions. In addition, the prevalence of determinants of DM was calculated, directly standardised to the age distribution of the total population. The association of determinants with DM was studied using univariate logistic regression analysis. All variables showing an association of p ≤ 0.25 for the Wald test were selected for the multivariate analyses. Stepwise multiple logistic regression was then performed to construct the optimal risk score for the occurrence of DM among Hindustani Surinamese, African Surinamese and Dutch. Criteria for entry into and exclusion from the model were a p-value for the likelihood ratio test of 0.05 and 0.10, respectively. A new risk score was developed to determine the probability of having DM with a logistic regression model using data that would be routinely available in general practice. Variables considered were ethnicity, biomedical parameters and disease history, e.g. age, BMI, waist circumference, resting heart rate, first-degree relative with DM, hypertension, history of CVD. The risk score is based on the sum of the score of the variables included in the full model (see additional file 1: Risk score for DM SUNSET.pdf).

Subsequently, we evaluated the performance of the risk score and compared it to the sets of screening criteria derived from current guidelines by calculating the area under the Receiver-Operator Characteristic curve (AUC) as a measure of diagnostic accuracy. Before analyzing the data, it was decided to consider an AUC of less than 0.60 to be poor, 0.60–0.75 to be moderate, and higher than 0.75 to be good.

The cut-off for the risk scores at which fasting plasma glucose screening was indicated, was chosen such that the sensitivity was approximately 80%, but not over. Additionally, we determined the specificity, the total population selected for screening, the prevalence in the screened population (the positive predictive value), and the number needed to screen to detect a case of DM (NNS).

Finally, we estimated the diagnostic accuracy of the risk scores for the detection of a new case of DM, to simulate a situation where persons with known DM are excluded from screening. This was done because, ideally, only previously unknown cases would have been used in the derivation of the risk score for screening for unknown DM. However, given issues of power to enable statistical modelling, it was decided to base the score on all cases and to then also estimate the diagnostic accuracy of the risk scores for newly detected DM.

We assessed the ability of a simplified version of our risk score, with points corresponding to the calculated odds ratio, to detect new cases of DM by calculating the number needed to screen to identify a new case of newly detected DM (NNS_new_).

To assess the validity of the risk scores, we used bootstrapping techniques to estimate a 'confidence interval' (bCI) around the estimated AUC of all risk scores in our population. We took 1000 random samples, with replacement, from the study population. At each step the parameters in the predictive models were calculated. We subsequently estimated the AUC for each of the models and calculated the 2.5 and 97.5th percentile to indicate the 'confidence interval'[20].

All analyses were performed using the the SAS package, version 9.1 (SAS Institute Inc., Cary, USA.).

## Results

### Characteristics of the population

Table 1 shows the characteristics of the study population by ethnic group. Compared to the Dutch, the Surinamese migrants tended to be congregated at the lower end of the socio-economic structure. People of Hindustani Surinamese origin had more abdominal obesity than the African Surinamese and Dutch.

**Table 1:** Characteristics of 35–60 year old Hindustani Surinamese, African Surinamese and ethnic Dutch participants

			**Hindustani Surinamese** **(n = 336)** median (IQR) or n (%)	**African Surinamese** **(n = 593)** median (IQR) or n (%)	**Ethnic Dutch** **(n = 486)** median (IQR) or n (%)
**Age**	(years)		44 (39–50)	43 (39–48)	48 (42–54)
**Sex**	(female)		187 (55.6)	400 (67.5)	242 (49.8)
**Level of education**	(high^a^)		34 (10.3)	116 (19.8)	175 (36.5)
**Level of profession**	(high^b^)		98 (32.9)	257 (48.9)	279 (58.3)
**Physical activity**	(at least 30 minutes 5× per week^c^)		166 (52.2)	315 (56.2)	306 (63.2)
**History of CVD**	(myocardial infarction and/or stroke)		21 (6.3)	13 (2.2)	10 (2.1)
**First degree relative with DM**			273 (81.3)	371 (62.7)	202 (41.7)
**BMI**	(kg/m2)		26.7 (23.8–29.6)	27.6 (24.5–31.5)	25.4 (22.9–28.3)
	(> 25 kg/m2, Hindustani Surinamese: > 23 kg/m2)		276 (82.1)	420 (71.0)	253 (52.1)
**Waist circumference**	(cm)		94.2 (85.9–101.0)	92.9 (83.1–102.1)	90.3 (81.5–100.3)
	(increased waist circumference^d^)		261 (77.7)	398 (67.2)	280 (57.6)
**Resting heart rate**	(resting heart rate in bpm)		72.5 (66.5–80.5)	72.0 (65.0–80.0)	68.5 (61.0–76.0)
	(resting heart rate ≥ 90 bpm)		29 (8.8)	46 (7.9)	21 (4.4)
**Dyslipidemia**					
HDL-cholesterol	(mmol/l)		1.2 (1.0–1.5)	1.4 (1.2–1.7)	1.4 (1.2–1.7)
	(< 0.9 mmol/l (35 mg/dl) and/or treated)		63 (18.6)	21 (3.5)	31 (6.4)
Triglyceride	(mmol/l)		1.2 (0.9–1.8)	0.8 (0.6–1.2)	1.1 (0.8–1.7)
	(> 2.8 mmol/l (250 mg/dl) and/or treated)		51 (15.2)	15 (2.5)	41 (8.4)
**Blood pressure**					
SBP	(mmHg)		124.0 (112.0–137.5)	125.0 (114.5–140.5)	121.5 (111.0–134.5)
DBP	(mmHg)		81.5 (75.0–89.0)	82.0 (75.5–91.0)	78.5 (71.0–86.0)
Hypertension	(> 140/90 mmHg and/or on anti-hypertensive therapy)		115 (35.9)	223 (38.0)	125 (25.7)
**Diabetes mellitus**	(fasting plasma glucose ≥ 7.0 mmol/l and/or self-reported)				
	in 35–44 years	known	17 (9.8)	21 (6.1)	5 (3.0)
		newly detected	12 (6.9)	7 (2.0)	2 (1.2)
	in 45–60 years	known	50 (30.7)	38 (15.4)	14 (4.4)
		newly detected	7 (4.3)	9 (3.6)	12 (3.8)
	Age standardised^e^	known	298 (21.1)	164 (11.6)	46 (3.2)
		newly detected	80 (5.7)	37 (2.6)	32 (2.2)

^a ^definition: higher vocational or more (i.e. primary, secondary or lower vocational versus higher vocational or more) ^b ^definition: grade of employment (as classified by the EGP scheme (routine non-manual employees or higher (high) vs. low) ^c ^Dutch physical activity guideline ^d ^waist circumference > 80 cm for women and > 94 cm for men ^e ^Directly standardised to the age distribution of the total population (in 5-year categories). Data represent expected number of cases (prevalence). BMI = body mass index, bpm = beats per minute, SBP = systolic blood pressure, DBP = diastolic blood pressure, CVD = cardiovascular disease, HDL = high density lipoprotein, DM= diabetes mellitus, IQR = interquartile range

### Prevalence and determinants of DM

Overall, Hindustani Surinamese had the highest prevalence of DM 25.6%, followed by African Surinamese 12.7% and Dutch 6.8%. The age-standardised prevalence among these groups was 26,7, 14,2 and 5,5, respectively. The prevalence of known and newly detected DM by age-group is shown in table 1. In the age-group 35 to 44 years, the sex-adjusted odds ratio (OR) for DM was 4.6 [2.0–10.9] for Hindustani Surinamese and 1.9 [0.8–4.6] for African Surinamese as compared to the Dutch. In the age group 45 to 60 years, the OR was 6.1 [3.7–10.3] for Hindustani Surinamese and 2.7 [1.6–4.6] for African Surinamese. Hindustani Surinamese and African Surinamese with DM had a higher odds of being detected, i.e. having known diabetes, than the Dutch (Hindustani Surinamese OR: 2.6 [1.1–6.2], African Surinamese OR: 2.7 [1.1–6.7]).

The determinants of DM in the risk score are shown in Table 2. History of CVD (OR 5.4 [2.9–10.3]) and waist circumference (OR 5.3 [3.2–8.6]) showed the strongest association with DM in the univariate analysis. In multivariate analysis, the strongest determinants were history of CVD (OR 3.0 [1.5–6.3]), a first-degree relative with DM (OR 2.7 [1.8–4.2]) and Hindustani Surinamese origin (OR 2.7 [1.7–4.5]).

**Table 2:** Determinants of diabetes mellitus among Hindustani Surinamese, African Surinamese and ethnic Dutch aged 35–60 years^a^

***Determinant***	***DM*** ***(n = 194)*** *Prevalence (%)*	***no DM*** ***(n = 1221)*** *Prevalence (%)*	***Univariate *** *OR [95% CI]*	***Multivariate*^b ^** *OR [95% CI]*
Age ≥ 45 years	67.0	49.0	2.2 [1.6–3.0]	1.8 [1.2–2.6]
BMI > 25 kg/m2^c^	89.1	63.6	4.8 [3.0–7.7]	1.9 [1.0–3.4]
Increased waist circumference^d^	89.6	62.7	5.3 [3.2–8.6]	2.3 [1.3–4.1]
Resting heart rate ≥ 90 bpm	15.4	5.6	3.1 [1.9–4.9]	2.4 [1.4–4.0]
First-degree relative with DM	82.9	56.2	3.8 [2.5–5.6]	2.7 [1.8–4.2]
Hypertension^e^	59.0	28.9	3.6 [2.6–4.9]	2.4 [1.7–3.4]
History of CVD	9.8	2.1	5.4 [2.9–10.3]	3.0 [1.5–6.3]
Ethnic group:				
Hindustani Surinamese	44.3	20.5	4.8 [3.1–7.4]	2.7 [1.7–4.5]
African Surinamese	38.7	42.4	2.0 [1.3–3.1]	1.5 [0.9–2.4]
Ethnic Dutch	17.0	37.1	1	1

^a ^only determinants selected for the multivariate analysis, based on a Waldtest for univariate association of p < 0.25 are shown ^b ^mutually adjusted ^c ^> 23 kg/m2 for Hindustani Surinamese ^d ^waist circumference > 80 cm for all women, > 94 cm for African Surinamese and ethnic Dutch men and > 90 cm for Hindustani Surinamese men ^e ^blood pressure > 140/90 mm Hg and/or being on anti-hypertensive therapy

DM = diabetes mellitus (fasting plasma glucose ≥ 7.0 mmol/l and/or self-reported), OR = odds ratio, CI = confidence interval, BMI = body mass index, bpm = beats per minute, CVD = cardiovascular disease (myocardial infarction and/or stroke)

### Performance of criteria for screening

Table 3 shows the performance of four sets of screening criteria in the three population subgroups. Selection based on age alone (set 1) showed the lowest diagnostic accuracy. Application of the risk score (set 4) resulted in a moderate to good diagnostic accuracy: the AUC was 0.74 (0.70–0.79) for the Hindustani Surinamese, 0.80 (0.75–0.85) for the African Surinamese, and 0.78 (0.73–0.85) for the Dutch, with a NNS of 3 among the Hindustani Surinamese, 5 among the African Surinamese, and 7 among the Dutch. Trends were similar when the analysis was restricted to persons with normoglycemia and newly detected DM (Table 4).

**Table 3:** Performance of sets of selective screening criteria for diabetes mellitus among participants in SUNSET

		***Source***	***AUC (bCI)*^c^**	***Sens (%, CI)***	***Spec (%, CI)***	***Screen (%)***	***Prev (%)***	***NNS***
**Set criteria for Hindustani Surinamese**								

1	age ≥ 45 years	DCGP	0.62 (0.58–0.67)	66.3 (55.2–75.9)	57.6 (51.2–63.8)	48.5	35.0	3
2	age ≥ 45 and BMI > 25 kg/m2	ADA	0.65 (0.61–0.71)	66.3 (55.2–75.9)	57.6 (51.2–63.8)	48.5	35.0	3
3	criteria 2, and have additional risk factors, as follows^a^	ADA extended	0.74 (0.70–0.79)	79.5 (69.0–87.3)	47.4 (41.0–53.8)	56.4	35.5	3
4	tailored risk score^b^	present study	0.74 (0.70–0.79)	75.9 (65,.0–84.3)	54.1 (47.6–60.4)	53.5	35.8	3

**Set criteria for African Surinamese**								

1	age ≥ 45 years	DCGP	0.62 (0.58–0.67)	62.7 (50.7–73.3)	61.4 (57.0–65.6)	41.7	19.0	6
2	age ≥ 45 and BMI > 25 kg/m2	ADA	0.68 (0.65–0.75)	59.5 (47.4–70.5)	72.6 (68.5–76.3)	81.1	14.2	8
3	criteria 2, and have additional risk factors, as follows^a^	ADA extended	0.79 (0.76–0.85)	78.1 (66.6–86.6)	60.7 (56.3–65.0)	44.1	22.1	5
4	tailored risk score^b^	present study	0.80 (0.75–0.85)	79.2 (67.7–87.5)	62.3 (57.9–66.5)	42.8	23.0	5

**Set criteria for ethnic Dutch**								

1	age ≥ 45 years	DCGP	0.57 (0.51–0.63)	78.8 (60.6–90.4)	35.6 (31.2–40.2)	65.4	8.2	13
2	age ≥ 45 and BMI > 25 kg/m2	ADA	0.72 (0.65–0.77)	72.7 (54.2–86.1)	72.7 (60.3–69.3)	79.8	8.0	13
3	criteria 2, and have additional risk factors, as follows^a^	ADA extended	0.79 (0.75–0.87)	71.9 (53.0–85.6)	71.2 (66.7–75.3)	31.7	15.0	7
**4**	tailored risk score^b^	present study	0.78 (0.73–0.85)	77.4 (58.5–89.7)	68.8 (64.2–73.1)	34.3	14.9	7

^a ^physically inactive or 1st-degree relative with DM or high risk ethnic group (Hindustani Surinamese, i.e. South Asian) or hypertensive or reduced HDL and/or elevated triglyceride or history of cardiovascular disease.

^b ^optimal screening criteria identified from multivariate analysis (ethnic groups, age, BMI, waist circumference, resting heart rate, first-degree relative with DM, hypertension, history of cardiovascular disease)

^c ^bCI: The 'Confidence interval' around the score was estimated via a bootstrapping procedure [20], the 2.5 and 97.5 percentiles are listed between brackets. The cut-off value for the SUNSET score was 0.200 for Hindustani Surinamese, 0.077 for African Surinamese and 0.055 for ethnic Dutch. DM = diabetes mellitus (fasting plasma glucose ≥ 7.0 mmol/l and/or self-reported), CI = 95%-confidence interval, DCPG = Dutch College of General Practitioners, ADA = American Diabetes Association, AUC = area under the curve, a measure of diagnostic accuracy, sens = sensitivity of the criteria (value closest to, but not over 80%), spec = specificity corresponding to the listed sensitivity, screen = percentage of the total population in this study that is screened, prev = prevalence in the screened population (the predictive value positive), NNS = number needed to screen to detect a case of DM in this study, BMI = body mass index

**Table 4:** Performance of sets of selective screening criteria for the identification of newly detected diabetes mellitus among participants in SUNSET^c^

		***Source***	**Hindustani Surinamese** ***N = 269*** **AUC (bCI)**	**African Surinamese** ***N = 534*** **AUC (bCI)**	**ethnic Dutch** **n = 467** **AUC (bCI)**
1	age ≥ 45 years	DCGP	0.53 (0.50–0.61)	0.59 (0.51–0.69)	0.61 (0.54–0.68)
2	age ≥ 45 and BMI > 25 kg/m2	ADA	0.61 (0.54–0.70)	0.69 (0.62–0.78)	0.72 (0.65–0.82)
3	criteria 2, and have additional risk factors, as follows^a^	ADA extended	0.69 (0.64–0.83)	0.87 (0.83–0.95)	0.80 (0.74–0.91)
4	tailored risk score^b^	present study	0.70 (0.66–0.83)	0.87 (0.83–0.93)	0.78 (0.73–0.90)

^a ^physically inactive or 1st-degree relative with DM or high risk ethnic group (Hindustani Surinamese, i.e. South Asian) or hypertensive or reduced high density lipoprotein and/or elevated triglyceride or history of cardiovascular disease.

^b ^optimal screening criteria identified from multivariate analysis (ethnic groups, age, BMI, waist circumference, resting heart rate, first-degree relative with DM, hypertension, history of cardiovascular disease)

^c ^Persons with known DM were excluded from the analysis. Data are area under the curve (AUC) and a 'confidence interval' (bCI), based on the 2.5 and 97.5 percentiles estimated via a bootstrapping procedure [20].

DM = diabetes mellitus (fasting plasma glucose ≥ 7.0 mmol/l and/or self-reported), DCPG = Dutch College of General Practitioners, ADA = American Diabetes Association

In Table 5, we listed the performance of the simplified version of the score (as specified in additional file 1: Risk score for DM SUNSET.pdf), among the population without a prior diagnosis of DM (known DM). The AUC varied between 0.58 among the Hindustani Surinamese to 0.79 among the African Surinamese. At a cut off of 8 points, 13 Hindustani Surinamese, 22 African Surinamese or 13 ethnic Dutch would have to be screened to detect a new case of DM.

**Table 5:** Performance of the simplified risk score for the identification of newly detected diabetes mellitus^a^

		***AUC (bCI)***	***Sens (%)***	***Screen (%)***	***Prev (%)***	***NNS*_new_**
**Hindustani Surinamese**		0.58 (0.49–0.70)				

	Total population				6.0	
	≥ 5 points		94.4	97.3	6.6	16
	≥ 8 points		94.4	80.7	8.0	13
	≥ 11 points		38.9	42.0	6.3	16

**African Surinamese**		0.79 (0.70–0.89)				

	Total population				3.1	
	≥ 5 points		93.8	86.8	3.3	31
	≥ 8 points		87.5	58.9	4.5	22
	≥ 11 points		81.3	25.8	9.6	11

**Ethnic Dutch**		0.77 (0.68–0.85)				

	Total population				3.1	
	≥ 5 points		100	56.2	5.5	19
	≥ 8 points		64.3	24.9	8.0	13
	≥ 11 points		14.3	6.4	6.9	15

^a ^Persons with known DM were excluded from the analysis. Simplified score is presented in additional file 1: Risk score for DM SUNSET.pdf. Cut off values (number of points) chosen based on the 20–50–75 percentiles in the total population.

DM = diabetes mellitus (fasting plasma glucose ≥ 7.0 mmol/l and/or self-reported), sens = sensitivity of the criteria at the given cut-off, screen = percentage of the total population that is screened at the given cut-off, prev = prevalence in the screened population (the predictive value positive), NNS_new _= number needed to identify a case of newly detected DM, i.e. a previously undiagnosed case of DM in this study, AUC = area under the curve, a measure of diagnostic accuracy, bCI = 'confidence interval' estimated via a bootstrapping procedure [20], the 2.5 and 97.5 percentiles are listed between brackets.

## Discussion

To our knowledge, this is the first European study outside the UK, to report on an evaluation of a risk score as a screening test for DM across different ethnic groups. The findings of our study confirm that DM varies strongly across ethnic groups; Hindustani Surinamese had the highest prevalence of DM, followed by African Surinamese and Dutch. Although the Hindustani Surinamese population seemed to have a higher proportion of known DM, the absolute prevalence of newly detected DM was still higher as compared to the other ethnic groups in both age categories. These results indicate that Hindustani Surinamese in particular, but also the African Surinamese, could benefit from screening starting before the age of 45 (i.e. the currently advised threshold).

The high prevalence of DM among the Hindustani Surinamese and African Surinamese is consistent with studies among South Asian and Afro-Caribbean populations in the UK [13-16]. Research in the 1990s suggested that for every known case there was another undiagnosed case of DM [4,21,22]. The proportion of newly detected DM in our study was lower. The proportion among the Dutch in our sample (nearly one-third) is in agreement with the UK and the recent data in the USA [23,24]. The proportion of newly detected DM among the Surinamese was lower. A higher awareness of the risk of diabetes among Hindustani Surinamese in Dutch clinical practice may contribute (at least in part) to this low proportion, particularly among those aged 45 years and older. The relatively low proportion of newly detected diabetes among African Surinamese has also been found in Afro-Caribbeans in the UK [25]. This may be linked to the high prevalence of hypertension among African origin populations, as clinicians may be triggered to test for elevated (fasting) glucose levels during check-ups for hypertension.

Despite the relatively small proportion of newly detected DM in Hindustani Surinamese persons, the absolute prevalence of newly detected DM is relatively high in Hindustani Surinamese, particularly among those aged 35 to 45 years. This emphasizes the importance and potential benefits of (selective) screening among young people of Hindustani Surinamese origin. More research is needed to find out whether screening below 35 years of age can be useful for Hindustani and African Surinamese and other South Asian and African origin people.

The performance of the new risk score, that was developed in this study, appears to be at least as accurate or even more accurate as a screening test for DM than other sets of screening criteria derived from current guidelines. Compared to the criteria based on age and BMI alone, inclusion of a number of additional parameters significantly improved the performance for both the South Asian and African ethnic groups. The parameters included waist circumference and resting heart rate. In our analyses, we found that waist circumference appeared to be superior for predicting the risk of DM when compared to BMI, even if we used a lower cut-off (BMI ≥ 23) in South Asians as recommended by the WHO [26]. Moreover, an elevated heart rate is known to be a risk marker for CVD and associated with an increased risk of DM [27,28]. However, despite a broad range of specific determinants, South Asian ethnic origin in itself remained one of the most important predictors.

The advantage of our risk score as compared to the extended ADA criteria is that it does not require invasive specimen collection. Previous prospective studies have also looked at diagnostic criteria based on non-invasive parameters and shown that the diagnostic accuracy was potentially good [29-31]. However, only one of these studies incorporated ethnicity, only 'black' (African American), but did not specifically assess the performance of the score with only clinical parameters (i.e. non-invasive) by ethnic group [30]. The two other studies did not include ethnicity as part of the risk score and were therefore not able to design and evaluate the criteria in a multi-ethnic population.

Before drawing a conclusion, this study has some limitations which should be discussed. First, the performance of screening criteria in a study population in which the model is developed, is known to often be too optimistic. However, the sub-group analyses and the results of the bootstrapping procedure indicate that our estimate of the performance of our risk score was valid.

Second, as in many surveys, the diagnosis of DM was based on a single fasting plasma glucose, which might have underestimated the true prevalence rates for DM as compared to a situation where an oral glucose tolerance test had been employed [32]. Studies show that over 30% of persons with DM are missed if the diagnosis is based on fasting glucose alone, suggesting that the total prevalence of DM may be higher than reported in our analyses [33-35].

Moreover, these studies showed that the agreement between the two methods is dependent on determinants such as on age and BMI [33,35]. This may affect the validity of our risk score. The performance of the score for the identification of persons with DM diagnosed by means of the oral glucose tolerance test may be insufficient, as our score was solely based on determinants associated with DM persons identified by the fasting plasma glucose measurement. Further studies will have to explore the validity of the risk score for the identification of DM diagnosed by means of the oral glucose tolerance test.

Third, our study is based on cross-sectional self-reported data. This could have biased the results of behavioural factors if, as part of treatment, persons with DM changed their lifestyle. This would imply an underestimation of how lifestyle-related determinants contribute to DM. In addition, the cross-sectional nature of the study prevented us from assessing the risk of incident DM.

Fourth, the participation rate among those invited for the study was 60%. Although this is reasonable for this type of study, selective non-response may influence the representativeness of the results, i.e. the generalisability to the original sample. In our study, only small differences were found in participation in the interview by gender, marital status, household composition and urbanisation. Unfortunately, no data were available on determinants of DM in this population. Therefore, we could not determine whether participants and non-participants were comparable with regard to the determinants and risk of DM. However, further comparison of participants and non-participants in the physical examination revealed no differences with regard to self-reported DM and self-rated health between these groups. This suggests that the study population may be largely representative for the entire sample.

## Conclusion

Management of DM – especially in ethnic groups at high risk – deserves a great deal of attention in the form of early detection and prompt treatment. In our study, the prevalence of DM was so high among Hindustani Surinamese and to a lesser extent among African Surinamese that universal rather than selective screening may be indicated. In any case, detection and treatment among these ethnic groups should not just focus on persons 45 years or older, as is advised in most guidelines, but also include persons under the age of 45 years. If a choice for selective screening is made, an ethnicity-specific approach is required. Our risk score, which includes ethnicity, may be relatively easy to use in clinical practice. We have shown that it is as accurate as or more accurate than screening criteria derived from current guidelines. However, we do recommend further validation of this new risk score in practice.

## Competing interests

The authors declare that they have no competing interests.

## Authors' contributions

NRB assisted in the design of the SUNSET study, carried out measurements, participated in the statistical analysis and drafted the manuscript. IGMV carried out the statistical analysis, drafted the manuscript and carried out the revisions. GM, carried out measurements and commented on the interpretation of the results and the drafts of the manuscript. RPK conceived and designed the SUNSET study and commented on the interpretation of the results and the drafts of the manuscript. FH, JBLH and RPJM contributed to the interpretation of the results and the drafts of the manuscript. KS conceived and designed the SUNSET study, contributed to the statistical analyses and participated in the writing and revisions of the manuscript. All authors read and approved the final version of the manuscript.

## Pre-publication history

The pre-publication history for this paper can be accessed here:



## Supplementary Material

Additional File 1 'Appendix: The risk score for DM from the population based SUNSET cohort'. Instructions on how to calculate the simplified risk score for diabetes mellitus from the population based SUNSET cohort. Click here for file
