# Supplementary material for: Prevalence of diabetes mellitus and the performance of a risk score among Hindustani Surinamese, African Surinamese and ethnic Dutch: a cross-sectional population-based study
Source: BMC Public Health. 2008 Aug 1;8:271. doi: 10.1186/1471-2458-8-271 (PMC2533321; doi:10.1186/1471-2458-8-271)
Supplement: Additional File 1 — 'Appendix: The risk score for DM from the population based SUNSET cohort'. Instructions on how to calculate the simplified risk score for diabetes mellitus from the population based SUNSET cohort. [file 1471-2458-8-271-S1.pdf]

APPENDIX: The risk score for DM from the population based SUNSET cohort

**Original risk score**[a]		**Characteristic**		**Simplified score for practice**	
Alpha	-1.638	Constant			
Beta1X1	0	age < 45		x1	0
	0.293	age ≥ 45 year			2
Beta2X2	0	BMI ≤ 25 kg/m2		x2	0
	0.317	BMI > 25 kg/m2[b]			2
Beta3X3	0	waist circumference ≤ 80 cm for all women and ≤ 94 cm for black and white European men		x3	0
	0.411	waist circumference > 80 cm for all women and > 94 cm for black and white European men [c]			2
Beta4X4	0	resting heart rate < 90 bpm		x4	0
	0.433	resting heart rate ≥ 90 bpm			2
Beta5X5	0	no first-degree relative with DM		x5	0
	0.497	first-degree relative with DM			3
Beta6X6	0	no hypertension		x6	0
	0.433	hypertension [d]			2
Beta7X7	0	no history of CVD		x7	0
	0.555	history of CVD			3
Beta8X8	0	ethnic	Ethnic Dutch	x8	0
	-0.084	group:	African Surinamese		2
	0.547		Hindustani Surinamese		3

[a] Estimated probability of having DM: $1/1 + e^{-(\text{alpha} + \text{Beta1X1} + \text{Beta2X2} + \text{Beta3X3} + \text{Beta4X4} + \text{Beta5X5} + \text{Beta6X6} + \text{Beta7X7} + \text{Beta8X8})}$

[b] > 23 kg/m2 dor Hindustani Surinamese

[c] >90 cm for Hindustani Surinamese men.

[d] blood pressure > 140/90 mm Hg and/or being on anti-hypertensive therapy.

DM = diabetes mellitus (fasting plasma glucose ≥ 7.0 mmol/l and/or being on anti-diabetic therapy), BMI= body mass index, bpm= beats per minute, CVD = cardiovascular disease (myocardial infarction and/or stroke)
